# 3D ultrasound-based determination of skeletal muscle fascicle orientations

**DOI:** 10.1007/s10237-024-01837-3

**Published:** 2024-03-26

**Authors:** Annika S. Sahrmann, Lukas Vosse, Tobias Siebert, Geoffrey G. Handsfield, Oliver Röhrle

**Affiliations:** 1 Institute for Modelling and Simulation of Biomechanical Systems, University of Stuttgart, Pfaffenwaldring 5A, 70569 Stuttgart, Germany; 2 Institute of Sport and Movement Science, University of Stuttgart, Allmandring 28, 70569 Stuttgart, Germany; 3 Auckland Bioengineering Institute, University of Auckland, 70 Symonds Street, Auckland, 1010 New Zealand; 4 Stuttgart Center for Simulation Science, EXC2075 - 390740016, University of Stuttgart, 70569 Stuttgart, Germany

**Keywords:** 3D ultrasound, Skeletal muscle architecture, Image processing, Pennation angle

## Abstract

Architectural parameters of skeletal muscle such as pennation angle provide valuable information on muscle function, since they can be related to the muscle force generating capacity, fiber packing, and contraction velocity. In this paper, we introduce a 3D ultrasound-based workflow for determining 3D fascicle orientations of skeletal muscles. We used a custom-designed automated motor driven 3D ultrasound scanning system for obtaining 3D ultrasound images. From these, we applied a custom-developed multiscale-vessel enhancement filter-based fascicle detection algorithm and determined muscle volume and pennation angle. We conducted trials on a phantom and on the human tibialis anterior (TA) muscle of 10 healthy subjects in plantarflexion (157 ± 7^∘^), neutral position (109 ± 7^∘^, corresponding to neutral standing), and one resting position in between (145 ± 6^∘^). The results of the phantom trials showed a high accuracy with a mean absolute error of 0.92 ± 0.59^∘^. TA pennation angles were significantly different between all positions for the deep muscle compartment; for the superficial compartment, angles are significantly increased for neutral position compared to plantarflexion and resting position. Pennation angles were also significantly different between superficial and deep compartment. The results of constant muscle volumes across the 3 ankle joint angles indicate the suitability of the method for capturing 3D muscle geometry. Absolute pennation angles in our study were slightly lower than recent literature. Decreased pennation angles during plantarflexion are consistent with previous studies. The presented method demonstrates the possibility of determining 3D fascicle orientations of the TA muscle in vivo.

## Introduction

Architectural parameters of skeletal muscle such as pennation angle provide valuable information on the muscle function since they can be related to the muscle’s force generating capacity, fiber packing, and contraction velocity (Lieber and Friden 2000). Changes in the pennation angle during dynamic contractions—the ’gearing’ phenomenon—can significantly increase the contraction velocity of the muscle belly compared to the fascicle contraction velocity (Eng et al. 2018). Muscle models thus rely on realistic muscle geometries, including pennation angles, to provide accurate predictions of muscle deformation and force development during muscle contractions (Röhrle et al. 2008; Seydewitz et al. 2019).

In vivo determination of pennation angles has often relied on 2D ultrasound imaging, which results in a two-dimensional in-plane angle. 3D pennation angles have often been assessed by diffusion tensor imaging (DTI) (Damon et al. 2002; Heemskerk et al. 2010; van Donkelaar et al. 1999; Hiepe et al. 2013), which is a magnetic resonance imaging (MRI)-based technique that determines the direction of diffusion of water molecules within tissues. This can be used to reconstruct muscle fascicle tracts based on the assumption that water diffuses preferentially along, rather than across, muscle fibers within fascicles. However, DTI comes with several drawbacks such as long acquisition times, lack of portability, and high cost. In addition, DTI scans on human subjects usually employ a resolution, which is not sufficient for segmenting muscles, which requires an additional anatomical scan, e.g., a T1-weighted scan, to segment the muscle and generate a mask as a muscle region of interest for tractography.

2D ultrasound is a widely-used and clinically established tool for investigating skeletal muscles which overcomes these drawbacks. Compared to MRI, ultrasound is less expensive, enables faster acquisitions and is portable. An ultrasound image is created by the transducer sending out sound waves into the tissue where the amount of reflected waves determines the grayscale level of the image. When the ultrasound probe is aligned with the muscle fascicles, skeletal muscles appear in a stripe pattern with the fascicles displayed in black and the connective tissue between fascicles in white. Several studies have investigated characteristics such as muscle thickness and pennation angle using ultrasound in healthy (Fukunaga et al. 1997; Darby et al. 2013) and pathological conditions such as cerebral palsy (Bland et al. 2011; Shortland et al. 2001), stroke (Gao et al. 2009), or Duchenne muscular distrophy (Jansen et al. 2012). In addition, ultrasound is used to detect changes in soft tissue deformation and pennation angle caused by external forces, such as muscle compression (Ryan et al. 2021; Wakeling et al. 2013).

Current approaches for automatic detection of pennation angle from 2D ultrasound images use a multi-scale vessel enhancement filter combined with a wavelet and radon transform (Rana et al. 2009), or deep residual networks (Cunningham et al. 2017; Zheng et al. 2021) and a Kalman filter (Zhang et al. 2015; Liu et al. 2019). In 2D, these techniques are reliable methods for determining pennation angle (Kwah et al. 2013). However, 2D pennation angles resulting from these techniques are projections of the 3D fascicle arrangement and lack full descriptions of the fascicle orientations in 3D space. Furthermore, determination of pennation angles from 2D images is highly dependent on the correct alignment of the ultrasound transducer (Bénard et al. 2009; Bolsterlee et al. 2016) as it should optimally lie in the fascicle plane. In addition, pennation angles show high variance within a muscle (Schenk et al. 2013) and regional differences in pennation angles may exist (Martin-Rodriguez et al. 2023) which are difficult to capture and record with 2D ultrasound.

To overcome these problems of 2D ultrasound, there are some approaches to reconstruct the 3D muscle volume and architecture from series of 2D ultrasound images. Muscle volume is a useful morphological parameter that can be used to explore relationships between age, training level, and growth processes in animal and human subjects (Siebert et al. 2017; Hanson et al. 2009). One approach for acquiring volumes from ultrasound imaging is 3D freehand ultrasound where the ultrasound transducer is equipped with position trackers, e.g., optical retro-reflective motion capture markers or a magnetic sensor. The sonographer holds this equipped transducer perpendicular to the muscle fascicles and scans along the muscle’s longitudinal axis so that the image position and orientation is known for each image frame, thereby collecting an image stack of cross-sectional images of the muscle, which can be reconstructed into a 3D volume. This technique has been validated using magnetic resonance imaging (MRI) for volume measurements of the TA muscle (Esformes et al. 2002), the triceps surae muscles in children with cerebral palsy (CP) (Barber et al. 2018), and the medical gastrocnemius (MG) muscle (Barber et al. 2009). It has further been applied in healthy muscles, e.g., vastus lateralis and MG muscles (Weide et al. 2017) and TA muscle (Raiteri et al. 2016), and in children with CP on the MG muscle (Noorkoiv et al. 2019; Schless et al. 2017; Fry et al. 2004).

Rana and Wakeling (2011) determined fascicle orientations from 2D images and simultaneously applied optical motion tracking to define 3D direction cosines from the 2D directions. This method relied on the correct orientation of the transducer and took up to 120 s for acquisition. In a later study (Rana et al. 2013), the method was employed for quantifying 3D architecture of the triceps surae. Here, the computed pennation angles differed from previously obtained angles from 2D images. This may be caused by the different method of the pennation angle definition, which is mostly defined as the angle between the fascicles and the aponeurosis; however, in Rana et al. (2013), the pennation angle is the angle relative to the major axis of the muscle. Other studies (Weide et al. 2017; Kurihara et al. 2005) used the reconstructed volumes of 3D freehand ultrasound protocols to determine fascicle lengths.

Within this project, we use a custom-designed automated motor driven 3D ultrasound scanning system to reconstruct volumes of the tibialis anterior (TA) muscle. From these images, we will determine muscle volume and pennation angle. We use a protocol with a short acquisition time so that the imaging of a whole muscle takes approximately 15–20 s. Determination of these parameters is not sensitive to the orientation of the ultrasound transducer. To validate our algorithm for tubular structures, we performed measurements on a custom-designed phantom. Subsequently, we apply the algorithm on human tibialis anterior muscles in vivo.

## Methods

### Fascicle phantom

**Fig. 1:**
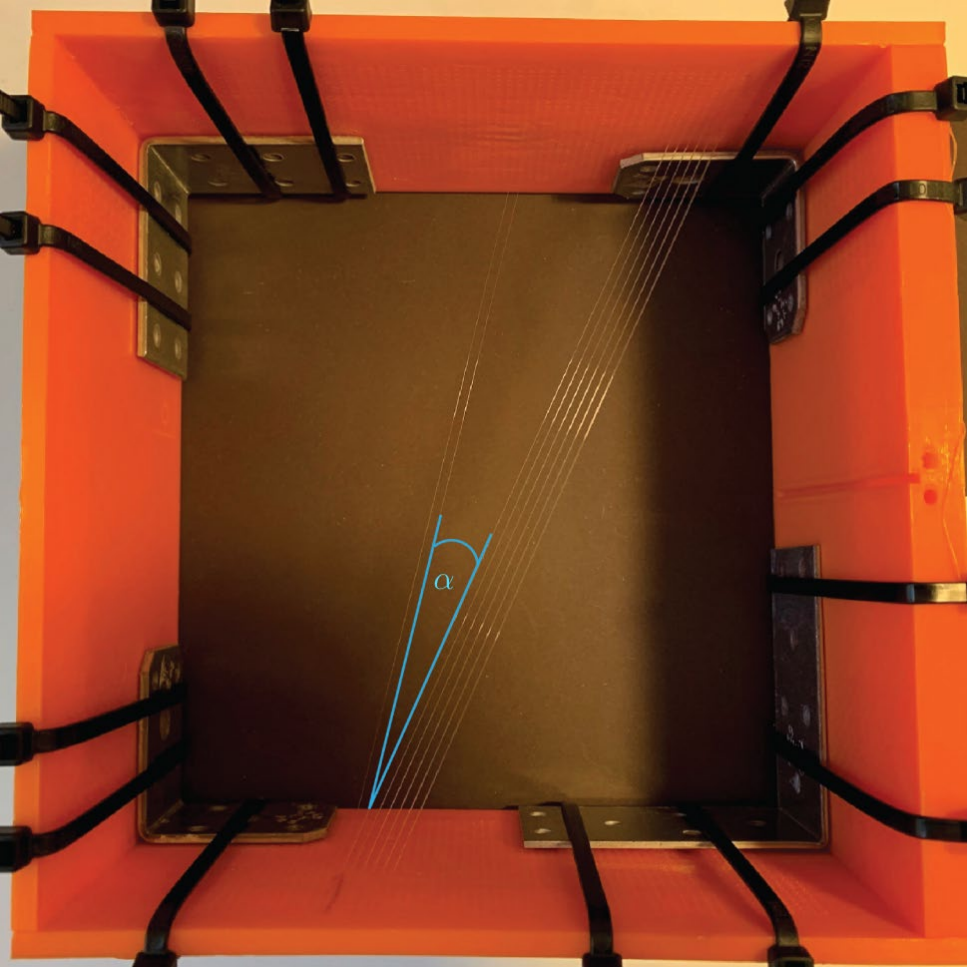
Fascicle Phantom with 0.1 mm nylon wires. The angle $\alpha$ between the wire compartments is displayed in blue

To investigate fascicle directions from 3D ultrasound, we designed and built a custom phantom. The fascicle phantom consists of four walls arranged in a square. The walls are 100 mm high, and each side is 200 mm wide. Two walls parallel to each other include 1680 holes (56 along the length and 30 along its height), each with a diameter of 1 mm. The distance between adjacent holes (in vertical and horizontal direction) is 2.5 mm. The value of 2.5 mm has proven to be effective in achieving both a small distance between holes and precise production. Wires were chosen based on the assumption that muscle fascicles can be described as tubular structure. Two wire groups are spanned from the holes of one wall to the holes in the parallel wall. One set of wires is arranged in a 3 rows $\times$ 6 columns array, using 18 holes on each opposite phantom wall. The other wire group consists of 2 wires horizontally side by side, resulting in 2 holes on each opposite phantom wall (Fig. 1). Thus, a total of 40 holes are used (2$\cdot$18 + 2$\cdot$2). In order to keep the wires tight, button-like elevations are attached to the outer sides of the phantom walls, to which the cords can be attached with rubber bands, similar to the PLUS toolkit N-Wire calibration phantom (Lasso et al. 2014). The wires were made from fishing line with a diameter of 0.1 mm, being the order of magnitude of the thickness of muscle fascicles. From this follows the assumption that the muscle fascicles can be described as tubular structures, which are well represented by the wires of a fishing line. Due to the known geometry of the phantom and its hole positions from the CAD model, the wire directions and the angle between the wire groups is known to be 10.78^∘^.

For ensuring a sufficient acoustic signal during ultrasound scanning, the phantom was immersed in a water tank.

### Data acquisition

#### Human subjects

We collected data from 10 healthy subjects (mean age, height, weight, body mass index (BMI) with standard deviation): 30 ± 4 years, 177 ± 8 cm, 69.3 ± 11.7 kg, 22.04 ± 1.98 $\frac{{{\text{kg}}}}{{{\text{m}}^{2} }}$, 6 male, 4 female). The experimental procedures involving human subjects described in this paper were approved by the University of Stuttgart’s Committee on Responsibility in Research (number: Az. 21-011).

#### Experimental trials

Cross-sectional images of the TA and fascicle phantom were taken by an ultrasound machine (Aixplorer MACH 30, SuperSonic Imagine, Aix-en-Provence, France) with a linear probe (SuperLinear SL18-5, SuperSonic Imagine, Aix-en-Provence, France). A frame grabber (USB3HDCAP, StarTech.com Ltd) recorded the ultrasound images with a frame rate of 30 Hz. The TA was chosen due to its superficial location and ease of detection with ultrasound. In addition, it plays a major role in walking as it is the strongest dorsiflexor in the lower limb. For moving the ultrasound probe, we used a custom-designed automated 3D ultrasound scanning system consisting of 3 moveable axes on which the transducer is moved along, of which two are driven by motors. Further, the system includes a force control mechanism. In this study, the force setting was set to a low force level (’1’), which has been experimentally determined to refer to a force of 4.1 N (Sahrmann et al. 2024). Taken together, these features enable the automated 3D ultrasound scanning system to perform controlled movement trajectories and consistent tissue deformations over the scanning length. The automated 3D ultrasound scanning system is further described in Sahrmann et al. (2024).

**Fig. 2:**
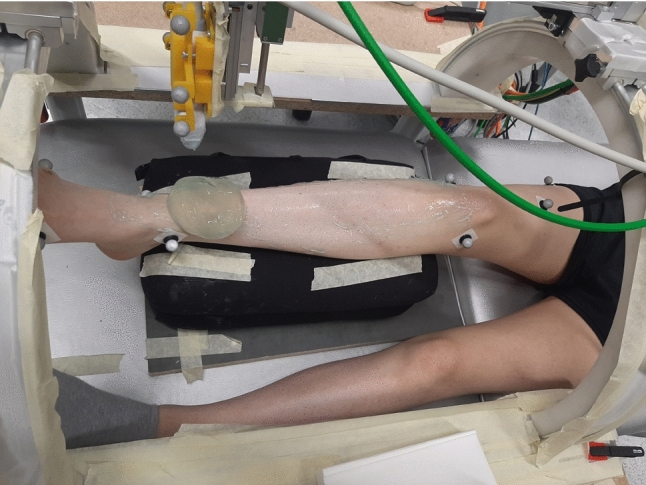
Subject setup with 3D ultrasound scanning system. The figure illustrates the resting position RP

For phantom trials, we recorded 11 trials of the fascicle phantom by scanning along the wires. For muscle trials, we obtained 3D ultrasound images of the TA muscle of the right legs of the 10 volunteer subjects. For this, the ultrasound probe is moved (in a linear direction) along the horizontal axis of the automated 3D ultrasound scanning system, such that the muscle’s CSA/the phantom wire groups are completely visible on each image. Thus, the probe is not aligned with the fascicle orientations while scanning. Since 3D ultrasound scans of the TA are possible with one single sweep, the angle of the circular axis remains constant during a sweep. Three ankle joint angles were examined. The subjects were asked to bring the foot into (1) maximum plantar flexion (PF) and (2) to a foot position corresponding to the ankle joint angle for neutral standing position (NP) (111 ± 2^∘^). One additional foot position (3) was examined, where the foot was in a resting position (RP), as illustrated in Fig. 2. The selection of NP as the foot position with the smallest ankle joint angle was due to the leg positioning, where the calf muscle was placed on a cushion and the foot and ankle are not supported. This setup restricted maximum dorsiflexion to the defined NP.

To record ankle angle positions, we placed optical reflective motion capture markers on the lateral and medial knee epicondyle, lateral and medial malleolus, first and fifth metatarsal, which were recorded by 8 infrared cameras (VICON, Oxford, UK). The ankle joint angle was computed as the angle between the vector from lateral knee epicondyle to lateral malleolus and the vector from lateral malleolus to fifth metatarsal. The mean joint angles for PF, RP, and NP were 157 ± 7^∘^, 145 ± 6^∘^, and 109 ± 7^∘^, respectively.

To avoid a loss of skin contact due to the natural curvature of the lower leg, we used a gel pad (Aquaflex, Parker Laboratories, Fairfield, USA) and ultrasound gel during scanning (Fig. 2). The gel pad was moved manually along the leg while scanning. The motor-driven ultrasound scanning device needs approximately 15 s to move from the distal to the proximal TA end. A total of 3 measurements were performed per subject (one for PF, RP, and NP, respectively).

### Calibration and 3D reconstruction

We used an N-Wire Phantom (Lasso et al. 2014) for determining the transformation between the ultrasound image pixel coordinate system and the 3D ultrasound device coordinate system. This process is called spatial calibration. For temporal calibration, the ultrasound probe was moved up and down in a water bath by the 3D ultrasound scanning device. Cross-correlating the line position in the image and the encoder data determines the time offset between them. We used a method for 3D reconstruction which has been described previously (Sahrmann et al. 2024). The method applies a series of coordinate transformations to transform the image pixels into a defined reconstruction volume, which is defined by applying a principal component analysis (PCA) on the image corner coordinates of the 2D image stack. The voxel sizes of the reconstruction volume are 0.17 $\times$ 0.17 $\times$ 0.66 mm and 0.14 $\times$ 0.14 $\times$ 0.37 mm, which means the distances between two consecutive images in the reconstruction volume are 0.66 mm and 0.37 mm for muscle and phantom data, respectively. The pixel values are assigned to the voxel positions in the map by using a nearest neighbor algorithm. Another nearest neighbor algorithm is used to assign values to empty voxels between voxels filled with image pixel values.

### Fascicle detection algorithm

**Fig. 3:**
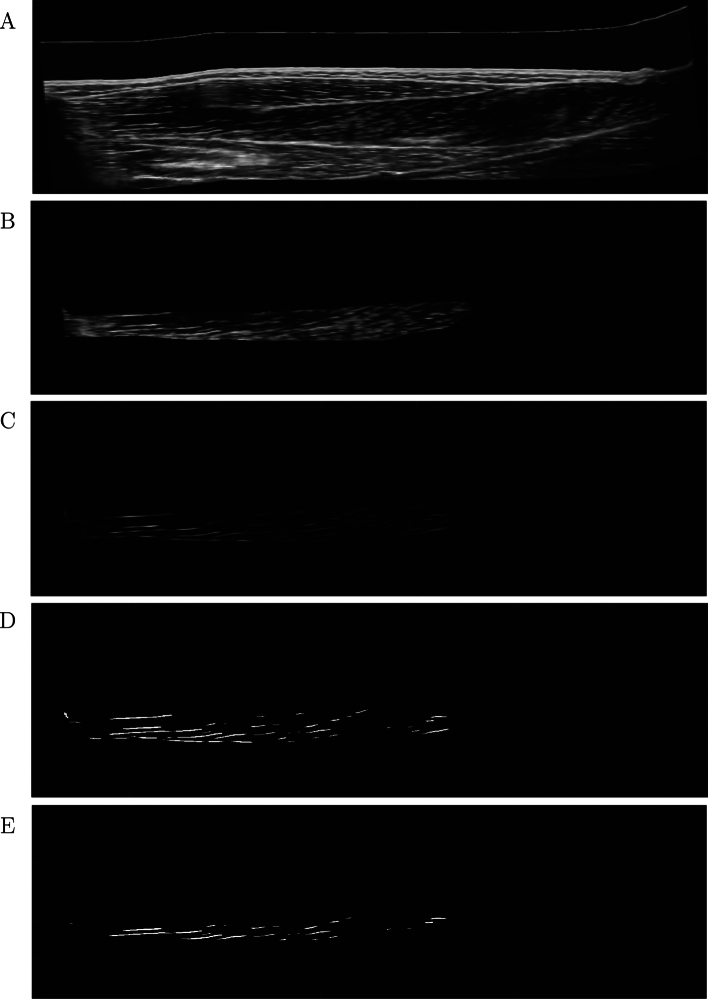
Workflow for fascicle detection from 3D ultrasound images. **A** Sagittal image slice of reconstructed volume rotated into muscle coordinate system, **B** masking of muscle compartment, **C** after applying MVEF, **D** binarizing filtered image and **E** shrunk masked

We used The Medical Imaging Interaction Toolkit (MITK) (Wolf et al. 2004) for semi-automatic segmentation of the reconstructed 3D volumes. In MITK’s segmentation tool, one manually outlines several boundaries of a geometry on a defined plane (on muscle data, this is the axial plane, i.e., the plane where the CSA can be segmented). The program includes an inbuilt interpolation algorithm for interpolation over the whole segmentation length, i.e., from the first to the last segmented slice, such that there are no empty slices within the segmentation. In case of phantom data, segmentation length means the first to last segmented slice, where the wires were visible on the image. The segmentation length for muscle data is the distance from the most proximal to most distal segmented CSA of the TA. For phantom data, both wire compartments were segmented separately. For muscle data, we segmented the whole TA as well as the superficial and deep compartments separately. We created geometries, i.e., surface models, of the muscle from the segmentation masks. We used MITK’s drawing tool for segmenting the aponeurosis. We used the segmentations to mask the 3D reconstructed images (Fig. 3B). The aponeurosis mask was used to avoid detection of aponeurosis directions and set all image values within the aponeurosis mask to 0.

For muscle data, we applied a PCA on the cartesian coordinates of the surface model of the whole muscle to determine the 3 principal axes of the muscle geometry. We took the 3 principal axis directions to establish a muscle coordinate system and rotated the masked reconstructed volume, so that it is aligned with the muscle coordinate system, i.e., that the sagittal plane of the 3D volume is aligned with the sagittal plane of the muscle coordinate system.

We applied a ’Hessian based Frangi Vesselness filter’ from *Matlab File Exchange* (Manniesing and Niessen 2005; Kroon 2021), which uses a multi-scale vessel enhancement filter (MVEF) (Frangi et al. 1998) on the 2D sagittal images (yz-plane for muscle, xz-plane for phantom data, Fig. 3C). The MVEF applies a Gaussian filter ($\sigma$=2 for muscle data and 3.5 for phantom data) to the image and computes the Hessian Matrix and their eigenvalues and eigenvectors to enhance the white lines on the images. As sensitivity threshold values (Frangi et al. 1998), we chose the function’s default values $\beta$ = 0.5 and *c* = 15. The filter settings were constant over all subjects. In this step of the algorithm, the purpose of employing the MVEF is to determine the voxel positions of the enhanced lines, i.e., the positions of the perimysium. The 3D fascicle orientations are computed at a later stage of the algorithm.

After applying the MVEF on the 2D sagittal images, we re-stitched the filtered 2D image slices to a 3D volume. We filtered the masked MVEF output with a Gaussian kernel and computed the Hessian matrix (2nd order gradients) of the Gaussian filtered volume. We computed the eigenvalues and eigenvectors of the filtered Hessian matrix and determined the eigenvector of the smallest eigenvalue for each voxel as the 3D fascicle direction. This means that each voxel contains a direction vector consisting of 3 components.

For determining the positions of detected fascicles, we binarized the re-stitched MVEF output volume by setting all voxels with a smaller value than 10 $\%$ of the maximum voxel value to 0 and all above to 1 and used the binary image as a mask for the direction fields (Fig. 3D). To determine this threshold of 10%, we examined the histogram of the voxel values and determined a value in the range of the most frequently occurring voxel values. We thus defined voxels with values below the threshold as erroneously detected fascicles. For muscle data, we shrank the mask of the compartment after MVEF in order to avoid detecting directions of the muscle’s surrounding epimysium and applied the mask to the filtered volume (Fig. 3E). The size of shrinking was adjusted to each subject and muscle compartment, where the average shrinking size was 10 pixels. In general, the shrinking size was higher for the superficial than for the deep compartment. We multiplied the direction components with the scaling factors of the volume in *x*-, *y*-, and *z*-direction. The scaling factors are the voxel dimensions, which means the size of each voxel in *x*-, *y*-, and *z*-direction (0.17 $\times$ 0.17 $\times$ 0.66 mm for muscle and 0.14 $\times$ 0.14 $\times$ 0.37 mm for phantom data).

In order to consider only the major orientations, vectors with a length smaller than 50% of the maximum direction vector length were removed. This threshold was achieved by manually testing the impact of different thresholds and selecting the one which visually smoothed the directions.

The detected directions at the end of the lines are detected in different directions around a sphere, as the algorithm detects tube or blob-like structures. Therefore, we removed all directions with less than 18 non-empty neighbors, in order to remove the end points of detected lines. The value 18 was selected in order to be able to remove enough incorrectly recognized directions (which are recognized as directions on a sphere) and still retain enough voxels at the correctly recognized fascicle orientation.

We applied a fast, unsupervised and robust discretized spline smoother from the literature (Garcia 2010a, b, 2023) on the detected directions to smooth the vector field, with the smoothing parameter set to 35. The smoother applies a penalized least square method. By using weights, the method is capable of working with missing values (NaNs). Thus, the smoother can be employed for inter- and extrapolation of the direction data. In order to fill the whole muscle volume with directions, we used a linear interpolation on the gridded vector field for muscle data. For extrapolating over the whole muscle volume, due to the earlier shrinking of the mask, we applied the spline smoother again with a very small smoothing parameter of 0.5.

The computed fascicle directions can be exported as a 3D vector field including position information in vtk format (Schroeder et al. 2006) after applying the fascicle detection algorithm. In this study, different filters of Paraview (version 5.8.0) are used to visualize fascicle tracts and directions.

### Pennation angle determination

#### Phantom data

We computed the directions at the detected positions after the fascicle detection algorithm for the two phantom compartments. From these directions, the angle was computed using the dot product formula. We computed the angle for each direction vector of one compartment with each direction vector of the other compartment.

#### Muscle data

For computation of the pennation angle, we applied a PCA on the points within the aponeurosis geometry and defined the largest principal component axis as the aponeurosis direction. Pennation angle was defined as the angle between the aponeurosis direction and the direction vector at each voxel. We computed the mean pennation angle overall volume elements of the muscle compartments at the three joint ankle positions (PF, RP, NP). To reduce the amount of data of the volume elements for easier computation and statistical analysis, we resampled the angles of the individual volume elements (from a magnitude of about 1$\cdot$10^7^) to 5000 per compartment.

All postprocessing of data such as fascicle detection and 3D reconstruction was done using Matlab (R2020a, MathWorks, MA, USA).

### Statistical analysis

We used a Shapiro–Wilk test for testing data for normal distribution. For examining significant differences between two different groups (differences between superficial and deep compartment), we used a Wilcoxon signed-rank test, as the data were not normally distributed. For examining significant differences between more than two different groups (differences between PF, RP, and NP), we used one way repeated measures ANOVAs for normally distributed data and Friedman tests for non-normally distributed data. The level of significance was set to *P* < 0.05. The effect sizes were classified as small (*r* = 0.1), medium (*r* = 0.3), and strong (*r* = 0.5) (Cohen 1988).

## Results

### Phantom study

**Fig. 4:**
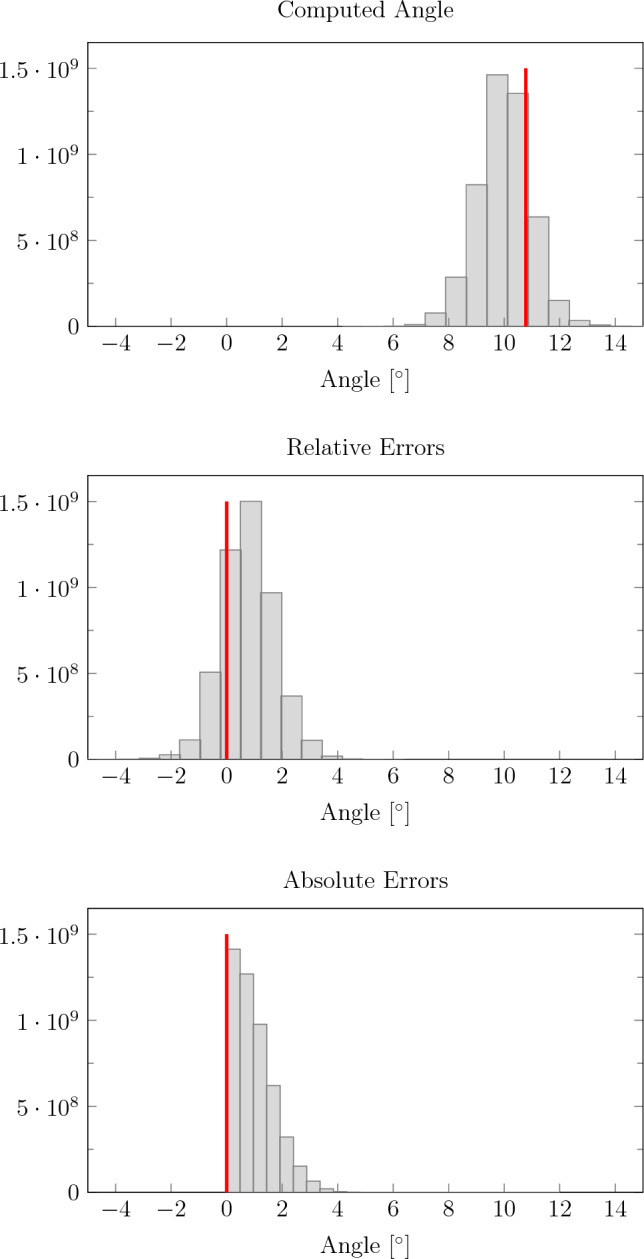
Computed wire phantom angles and absolute and relative errors. The phantom angle of 10.78^∘^ between the wire groups is shown as a vertical red line in the top subfigure

Figure 4 shows a histogram of the angle for each direction vector of one compartment with each direction vector of the other compartment. For comparing all directions, we found a mean absolute error of 0.92 ± 0.59^∘^ and a mean relative error of 0.80 ± 0.74^∘^.

We also computed the mean direction for both compartments as the normed sum of vectors and found a mean angle of 10.10 ± 0.35^∘^ and a mean absolute error of 0.68 ± 0.35^∘^.

### Muscle volume reconstructions

The computed mean muscle volume of the TA is 91.2 ± 23.5 cm^3^. Muscle volume did not differ significantly across the 3 ankle joint angles (*p* < 0.05), where muscle volumes at PF and NP were larger by 2.4 ± 6.0$\%$ and 1.6 ± 6.0$\%$, respectively, compared to RP.

### Muscle fascicle reconstructions

**Fig. 5:**
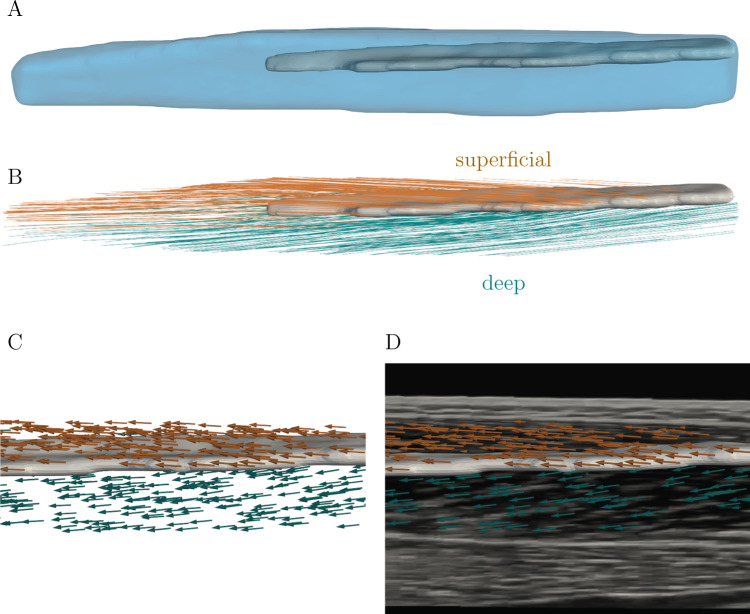
Fascicle reconstruction of the TA for one representative subject using Paraview. The superficial compartment is visualized in orange and the deep compartment in green, the aponeurosis is gray. **A** TA volume, **B** fascicle lines of the whole muscle using the Streamline Tracer filter, **C** fascicle directions as arrows using the Glyph filter, **D** overlay of a 2D sagittal image with the reconstructed fascicle directions, which are visually aligned

The 3D volume of the TA and its central aponeurosis obtained from segmentation and surface creation using MITK is illustrated for one representative subject in Fig. 5A. Figure 5B shows a visualization of the TA fascicle reconstructions after tractography using Paraview in neutral foot position, where superficial and deep compartment are illustrated in orange and green, the aponeurosis geometry is colored in gray. The figure shows that the fibers for the two compartments run in different directions from the central aponeurosis. Figure 5C illustrates the exported vector field from the fascicle detection algorithm as arrows. Figure 5D displays an overlay of the arrow visualization with a sagittal image slice of the reconstructed 3D ultrasound image, where the perimysium is shown as light gray or white lines between the black or dark gray-colored muscle fascicles. From visual inspection, the computed directions are aligned with the perimysium. Since the perimysium is parallel to the fascicles, this indicates that the computed directions are also aligned with the fascicles on the 3D volume image slice.

**Fig. 6:**
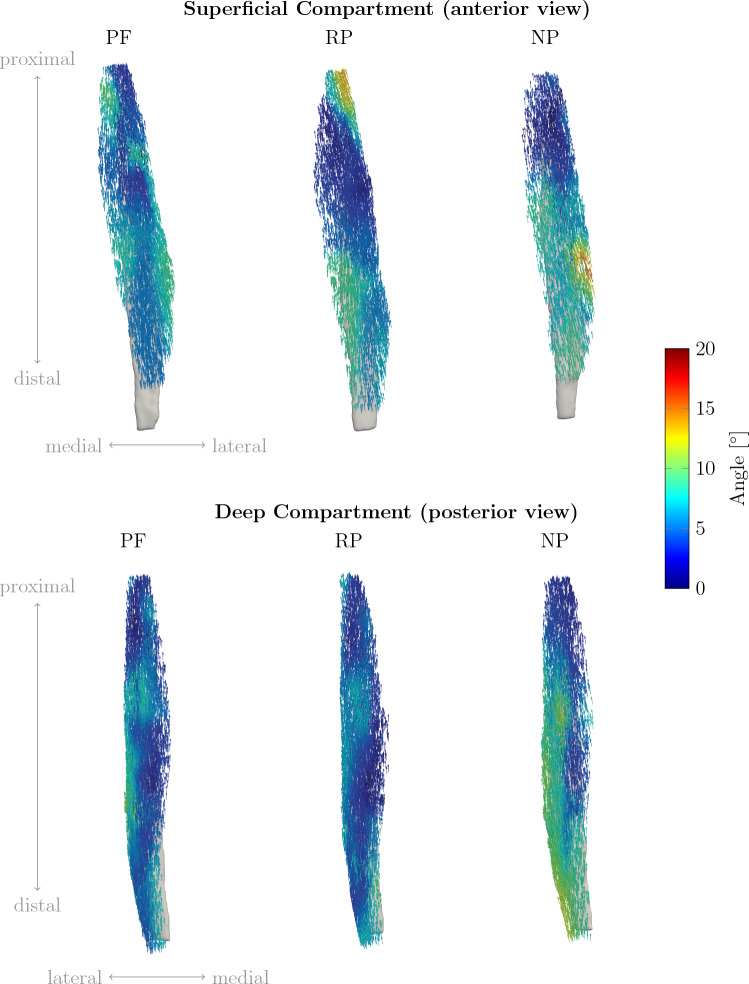
Fascicle direction distribution of one representative subject (the same as in 5) for superficial and deep compartment in PF, RP, and NP. The directions are color coded according to the computed pennation angle. For the NP, an increase in pennation angle can be observed especially in the distal part of the TA

In Fig. 6, the pennation angle for each voxel is displayed color-coded according to the magnitude of the angle for both compartments for PF, RP, and NP for the same subject as in Fig. 5. For the superficial compartment, pennation angle increases for NP are mainly in the distal and mid part of the muscle. For the deep compartment, pennation angle increase can be observed in the distal part at the lateral side of the muscle.

**Fig. 7:**
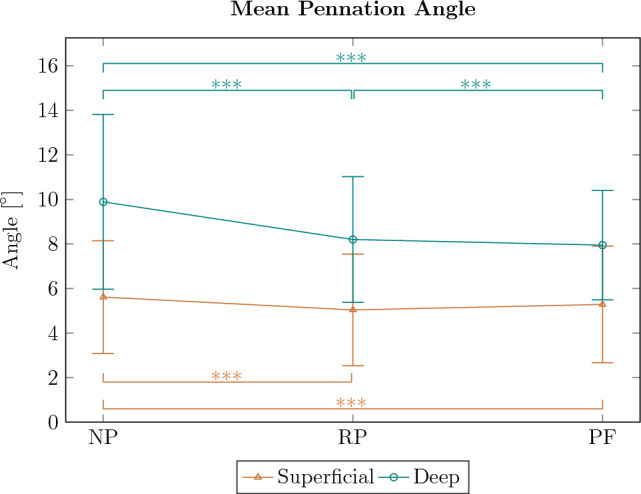
Average pennation angle for all subjects for superficial and deep compartment of the TA. The mean angles are increased for NP position compared to RP and PF, mean angles for the deep compartment are significantly increased compared to the deep compartment (*** indicates significant differences)

In both compartments, the differences between PF and RP are relatively small (about 1^∘^, Fig. 7). For the superficial compartment, pennation angles are significantly increased for NP, compared to RP and PF (*p* < 0.001 and effect size *r* = 0.0037 and *r* = 0.0033, respectively), whereas RP and PF do not show significant differences. For the deep compartment, the pennation angles for NP are significantly larger than for RP and PF (*p* < 0.001 and effect size *r* = 0.0072 and *r* = 0.008, respectively), also PF and RP show significant differences (*p* < 0.001 and effect size *r* = 0.0017). Overall, mean pennation angles are significantly smaller for the superficial than for the deep compartment (*p* < 0.001 and strong effect size *r* = 0.67).

## Discussion

In this paper, we provided a method for volume and 3D fascicle investigations of skeletal muscle. In terms of portability, shorter acquisition times and therefore enabling 3D scans during active contractions, this method shows advantages compared to other imaging methods such as MRI-based methods. We demonstrated the possibility of determining TA fascicle orientations in vivo from images obtained using an automated motor driven 3D ultrasound scanning system.

In a first step, we validated the method on a wire phantom with known fascicle orientations. The results of the phantom validation indicate a high accuracy of the proposed method for tubular structures. In a second step, we applied the method to 3D fascicle detection on a human TA, which requires further validation in future works. Furthermore, we were able to determine 3D muscle shape and muscle volume using the method.

The reproducibility of muscle volume measurements for different muscle lengths, corresponding to the 3 ankle joint angles, indicates the suitability of the method for capturing 3D muscle geometry (Böl et al. 2013). In our study, we found a mean TA muscle volume of 91.2 ± 23.5 cm^3^ for healthy subjects. These results are consistent with previous studies of healthy subjects using MRI [muscle volume 86.5 ± 18.7 cm^3^, 26 ± 2 years old, BMI 22.6 ± 2.7 (Jensen et al. 2016)]. Results of some cadaver studies show lower TA volumes using water displacement [65 ± 5 cm^3^ (Sopher et al. 2016)] and determination of the muscle volume via the muscle mass and muscle density $\rho =\text {1.056}\frac{\text {g}}{\text {cm}^\text {3}}$ [80.1 ± 26.6 g corresponding to 75.9 ± 25.2 cm^3^ (Ward et al. 2008)]. This can be explained by the fact that their subjects were older (80 and 83 years, respectively) than the average age of our subject group (29.9 ± 4.43 years). Other previous studies however found larger TA volumes using MRI on healthy subjects [129 ± 22 cm^3^, 27 ± 4 years, 175 ± 10 cm, 76 ± 12 kg (Charles et al. 2019); 131.8$-$133.6 cm^3^, 23 ± 3 years, 175 ± 8 cm, 70 ± 6 kg, (Esformes et al. 2002)], or 3D ultrasound on healthy subjects [142–284 cm^3^, 24 ± 2 years, 175 ± 7 cm, 69 ± 10 kg (Raiteri et al. 2016)]. The differences of TA volume of the individual studies can have a variety of causes. Muscle volume can differ depending on the amount of movement, the type of sport and the training condition of the test subjects (Hanson et al. 2009). Moreover, in segmentation-based volume determinations [e.g., Barber et al. (2009), Sahrmann et al. (2019), Fry et al. (2004), Fukunaga et al. (1997), Raiteri et al. (2016), Cenni et al. (2018), Schless et al. (2017), Weide et al. (2017)], as used in most imaging methods, the calculated volume heavily depends on the segmentation quality and the segmenting operator. In order to maximize consistency and avoid differences in segmentations due to different operators, all segmentations in our study were done by the same operator. In addition, in manual segmentation a clear demarcation of adjacent muscles is not always possible (Fukunaga et al. 1992).

In our study, the determined pennation angles of the TA are relatively small, with angles of about 4^∘^ to 8^∘^ at neutral ankle joint angle (Fig. 7), which is slightly lower compared to literature data [e.g., 7^∘^ (Liu et al. 2014), 8^∘^ (Choi et al. 2019), 10^∘^ (Hagoort et al. 2022), 11^∘^ (Maganaris et al. 1998), 9–11^∘^ (Raiteri et al. 2018)]. One possible explanation for this decrease in pennation angle could be the constant, sensor-controlled contact pressure of the ultrasound probe of our device causing tissue deformation. Wakeling et al. (2013) showed that compression of the human medial gastrocnemius muscle with elastic bands leads to a reduction of the pennation angle. Similarly, it has been shown that increasing transverse contact force of an ultrasound sensor results in a reduction of the pennation angle (Ryan et al. 2019; Stutzig et al. 2019). The tibia provides a solid structure that can limit the tissue deformation of the TA muscle to some extent. Deformation in other muscles may therefore be greater than in the TA. However, the angle of the transducer relative to the tibia is a crucial factor in determining the amount of muscle tissue deformation that can occur with 3D ultrasound of the TA. In this study, the transducer is positioned at an angle from the side in order to image the entire muscle cross-section on each 2D image, which may cause tissue deformation. Therefore, the decreased absolute values of the pennation angles may be attributed to TA muscle tissue deformation.

The applied contact force in this study was 4.1 N. In a previous study (Lee et al. 2019) investigating contact forces on the TA muscle, significant differences in muscle thickness have been found for applied force levels of 1–4 N. Furthermore, significant changes in muscle thickness have been found for the transversus abdominis for forces below 2 N (Ishida and Watanabe 2012). Both studies revealed changes in muscle thickness for force values below the force level employed in this study. Consequently, these changes in muscle thickness might result in a decrease of pennation angle. For investigating this effect more in detail, future studies could examine if there are differences between low forces and no contact force at all by using a water tank. Possibly, this influencing factor could be prevented by contact less measurement of muscle architecture with an ultrasonic sensor in a water bath.

Furthermore, we see the nature of the method for determining the fascicle orientation as another reason for smaller pennation angles than in previous literature. In a study by Rana et al. (2009), fascicle orientations of the vastus lateralis muscle were determined from 2D ultrasound images using a wavelet transform, Radon transform and manual digitization. The determined pennation angles were found to be smaller for the wavelet transform than for the Radon transform or manual digitization in some images. The authors justified this by stating that manual determination of fascicle directions is more likely to be based on prominent, longer fibers (the Radon transform is similar). However, the wavelet transform considers the mean of local orientations within a defined kernel size, which is also relevant for investigating the fascicle curvature (Rana et al. 2014). Considering this, the method of our study also incorporates more local orientations through voxel-wise eigenvalue decomposition, rather than relying on prominent lines as in manual determination (and in the aforementioned studies with larger average angles).

A large number of studies show a decrease in the pennation angles with muscle lengthening from NP to PF [pennation angle decrease from: 8^∘^ to 6^∘^, with 45^∘^ range of motion between NP and PF (Liu et al. 2014), 10^∘^–7^∘^, with 30^∘^ range of motion between NP and PF (Hu et al. 2019), 12^∘^–9^∘^, with 30^∘^ range of motion between NP and PF (Maganaris and Baltzopoulos 1999), 10^∘^–8^∘^, with 30^∘^ (0.52 rad) range of motion between NP and PF (Reeves and Narici 2003) 10^∘^–9^∘^, with 15^∘^ range of motion between NP and PF (Raiteri et al. 2018)]. These studies measure the 2D pennation angles of some representative fascicles in the middle region of the muscle or their compartments. With our 3D characterization, we also found a significant decrease in the pennation angle for the deep TA region with muscle lengthening from NP over RP to PF (Fig. 7). The superficial region shows significantly smaller changes in the pennation angle with muscle lengthening, becoming significant only between the NP and RP as well as between NP and PF. The low effect sizes and the comparatively large standard deviations of the pennation angles might be attributed to methodological differences between our study and classic 2D ultrasound studies. 3D characterization with much higher spatial resolution compared to local pennation data from 2D ultrasound lead to comparable large variability of the pennation angles within the muscle (Fig. 7). Nevertheless, studies agree that there are only small changes in pennation angle with muscle lengthening in the TA. This can be explained by a geometrical model of Eng et al. (2018), in which, depending on the muscle deformation during muscle lengthening, there is no or negligible change in the pennation angle. In a study from 2018, Raiteri et al. (2018) found that the longitudinal stiffness of the central aponeurosis in the TA increases with muscle lengthening. This in turn yielded a decrease in TA muscle fascicle shortening, which directly impacts the change of pennation angle in pennate muscles. Moreover, the decrease in absolute values of the pennation angle might also cause smaller differences in the pennation angle for different ankle joint angles. This could be due to tissue compression or the nature of the method, as discussed in the previous sections. In addition, we found significantly higher pennation angles in the deep compared to the superficial region (Fig. 7). This agrees with measurements on the TA of women (*n* = 45), but is in contrast to men (*n* = 64) (Martin-Rodriguez et al. 2023). In our study, due to the small number of participants (6 male, 4 female), we cannot examine these differences statistically.

In this study, we addressed the determination of the fascicle direction, i.e., the pennation angle. Due to the contraction behavior and physiology of skeletal muscles, other fascicle parameters, such as fascicle length or curvature, are also important determinants of the muscle’s function, which makes the evaluation of such parameters relevant. Thus, one limitation of our study is the absence of these parameters, since the algorithm so far computes solely fascicle orientations. Therefore, future work should focus on the development of advanced tractography algorithms similar to the ones for processing DTI data sets, where the course of fascicles can be tracked throughout the 3D voxel array. With this, fascicle length and curvature could be determined as well.

We employed the fascicle tracking method on the TA, for demonstration purpose. The way the device of the automated 3D ultrasound scanning system is designed, it is in general possible to be used on other muscles, as it would require the examined body part to be aligned underneath the semicircles. However, one limitation of ultrasound is its imaging depth and the fact that sound waves cannot penetrate tissue such as bone, as the waves are reflected at such tissue boundaries. This leads to a black spot in the image behind the reflection border. The method presented in this study is therefore mainly suitable for superficial muscles and less for deeper muscles. In addition, care must be taken to position the transducer relative to the bone during examinations. After reconstruction of the 3D ultrasound data set of other superficial muscles, the fascicle tracking algorithm can be applied as well and parameters might need to be adjusted. Since we segmented the superficial and the deep compartment separately, this would need to be done with other non-fusiform muscles as well. For some muscles, where the compartments are not easily recognizable on the cross-sectional area, this can be challenging. Therefore, the application on other muscle also depends on the architecture of the muscle and its appearance on the ultrasound image.

In this study, we used a custom-designed wire phantom for evaluating the presented method. It should be noted that the success of the method on this phantom does not necessarily ensure the success of its application on muscle tissue. Since the phantom wires are tubular structures, the method is validated for such ideal tube-like structures. By applying this method to muscle data, we assumed that muscle fascicles are rather arranged in tube-like structures. To further investigate the transferability of the phantom validation results to muscle data, future studies should focus more on the arrangement of the fascicles, i.e., if they are arranged in tubes or sheets. Furthermore, validation studies on muscle tissue should be carried out, possibly ex vivo using manual digitization (Siebert et al. 2015).

## Conclusion

In conclusion, we presented a method that allows the possibility of 3D reconstruction of 3D skeletal muscle fascicle orientations and volumes in young healthy subjects. Validation of the method on phantom data may indicate a high accuracy of the method for ideal tube-like structures. However, future work requires further evaluation and validation of the method applied to muscle tissue, e.g., by comparison with other state-of-the-art imaging techniques such as DTI or, as mentioned above, ex vivo by dissection. Moreover, at this stage the method provides measures of the pennation angle. As previously stated, other parameters such as fascicle length and curvature are also significant determinants (Papenkort et al. 2020), therefore it is important to obtain such parameters as well. Thus, the development of advanced tractography algorithms similar to those employed in DTI processing is needed for determination of these fascicle parameters.

With these extensions, the method presented in this study may be applied to clinical populations, as a less complex method for investigating 3D fascicle architectures.

In addition, the fascicle orientations obtained from our method can help improving of computational models of skeletal muscles (Seydewitz et al. 2019; Röhrle et al. 2016), thereby increasing the understanding of a muscle’s mechanical behavior with different fascicle orientations.
